# Dietary Exposure and Health Risk Assessment of Polycyclic Aromatic Hydrocarbons in Black Tea Consumed in Taiwan

**DOI:** 10.3390/toxics12020134

**Published:** 2024-02-07

**Authors:** Drewyan Minelly Harrison, Wei-Chung Chang, Hsin-Tang Lin

**Affiliations:** 1 International Master Program of Agriculture, National Chung Hsing University, Taichung 402-202, Taiwan; drewyanmharrison@gmail.com; 2 Graduate Institute of Food Safety, National Chung Hsing University, Taichung 402-202, Taiwan; weichung871227@gmail.com; 3 Department of Food Science and Biotechnology, National Chung Hsing University, Taichung 402-202, Taiwan; 4 Department of Law, National Chung Hsing University, Taichung 402-202, Taiwan

**Keywords:** *Camellia sinensis*, polycyclic aromatic hydrocarbons (PAHs), risk assessment, margin of exposure (MOE)

## Abstract

Polycyclic aromatic hydrocarbons (PAHs) are organic compounds found in many foods and drinks, and there have been some concerns over these compounds due to their carcinogenic nature. This study evaluated the concentrations of PAH4 (BaP, BaA, BbF, and CHR) in different black tea infusions and drinks based on the origin of the tea. The release of PAH4 from tea leaves to tea infusions was significantly low, with the highest transfer being 25.81%. The mean concentrations of BaP and PAH4 in tea infusions were used to conduct a risk assessment for the Taiwanese population, which showed that the 19–65 age group had the highest estimated intake of PAH4 and BaP among all age groups. These results, however, also showed margin of exposure (MOE) values well above the benchmark of 10,000. This indicated that PAH exposure from black tea consumption for the Taiwanese population constitutes a low-level health concern.

## 1. Introduction

Black tea, along with most other teas, is produced from the *Camellia sinensis* plant, with the final product being dependent on the manufacturing process. It is a fully fermented tea and is one of the most popular types of tea around the globe [1]. Taiwan is well known for its tea, which holds an important position in the culture of the country. In 2022, the per capita consumption of tea in Taiwan was 1.6 kg, with tea commonly being consumed hot with no additives such as sugar. Worldwide, the production of tea was estimated to be 6.5 million tons [1]. In 2021, the production of tea in Taiwan was only 11,900 tons [2]. As well producing its own tea, Taiwan also imports tea, with over 30,000 tons being imported yearly. The majority of black tea imports come from Vietnam and Sri Lanka. Vietnam is the largest exporter of tea to Taiwan, making up over a third of the imports. In 2021, Taiwan imported over 18,000 tons of tea from Vietnam [3].

Many tea shops are also found around the country, which are popular among the younger generations. These shops offer a large selection of teas with many additions such as coconut jelly, aiyu jelly, grass jelly, and tapioca pearls (commonly known as boba). Tea with boba or bubble milk tea is popular throughout the country and has become synonymous with Taiwan worldwide. It can be made with green and oolong tea but is more commonly made with black tea. Black tea is a popular type of tea which is more oxidized and goes through more processing. Black teas in general are popular as they contain many beneficial compounds, such as phenolic acids and aromatic compounds [4].

Despite its many benefits, studies have also found tea to be contaminated with heavy metals, pesticides, dioxins and PAHs [5,6]. Lin et al. (2005) [7] found that during the heating stages of the tea leaves, PAHs are formed. The oxidation process may also help bring out the PAHs developed from the heating processes. During food processing, high-temperature cooking processes such as grilling, smoking, frying, steaming, roasting, and drying also plays a role in the production of PAHs.

PAHs are a large category of organic compounds containing two or more fused benzene rings [8]. These substances arise from the incomplete combustion of carbon-based materials [9]. PAHs are formed through various processes including pyrolysis and pyro-synthesis. Pyrolysis refers to the thermal decomposition of organic materials at high temperatures, which often occur in a chemically inactive atmosphere. During pyrolysis, organic matter becomes partially decomposed and divides into unstable fractions. During pyrosynthesis, these unstable fractions are then recombined into reactive radicals, which are stable PAHs [10].

PAHs are resistant to degradation, allowing them to persist in the environment for extended periods. In 2008, the European Food Safety Authority (EFSA) has identified 16 PAHs which may be found in foodstuffs and can negatively affect health. These PAHs are known as European Union (EU) priority PAHs [11]. In 2011, the EFSA identified the sums of benzo[a]pyrene (BaP), benz[a]anthracene (BaA), benzo[b]fluoranthene (BbF), and chrysene (CHR) (known as PAH4) as suitable indicators for the occurrence of PAHs in food. These PAHs may be found in food and have the possibility of negatively affecting health. Concern over PAHs have increased in recent years due to their possibility of causing adverse effects on human health [12]. Scientific studies have linked PAHs to various health issues, including cataracts, kidney disease, and liver disease. There are many health concerns associated with PAH-contaminated food, however, the most important health concern is cancer. PAHs make up an extensive group of chemicals which are known to be carcinogenic. However, certain PAHs do not possess direct carcinogenic properties but may act as co-carcinogens, as their metabolites have the potential to induce cancer.

Studies have demonstrated the genotoxicity and mutagenicity of PAHs, especially BaP and its metabolites. BaP and other mutagenic PAHs are metabolized and form highly reactive compounds that can form DNA adducts with a high genotoxic/mutagenic potential or produce oxidative DNA damage, such as PAH quinones [13,14]. In addition, PAHs can react with trace amounts of NO_2_ and HNO_3_ to generate nitro-PAHs. Such nitro derivatives are also highly mutagenic [15,16,17]. According to the International Agency for Research on Cancer (IARC), agents can be classified based on their carcinogenic nature. In PAH4, BaP has been identified as a Group 1 agent which is carcinogenic to humans. BaA, BbF, and CHR are classified as Group 2A agents which are probably carcinogenic to humans [18].

The maximum levels of PAHs have been regulated in many foodstuffs by international organizations. The EFSA has established guidelines for PAHs in food stuff such as vegetable oils, seafoods and grains [11]. These standards are based on the sum of BaA, CHR, BbF and BaP which serve as a global reference and help individual countries establish their national regulations. In the context of food, the U.S. Food and Drug Administration (USFDA) has not established specific regulatory standards governing the allowable levels of PAHs in food products [19].

The Taiwan Food and Drug Administration (TFDA) has had set maximum limits for PAH4 in various food items since 2019 [20]. It was advised for the food industry to refer to the guidelines to reduce PAHs content through process optimization and quality control. These guidelines would assist in preventing or improving products that may contain traces of PAHs due to processing [21]. Proposed quality control indicators and action guidelines would serve as a reference for the industry. There are currently still no specified maximum limits for BaP in tea [20].

In Taiwan, the total amount of imported tea leaves has exceeded the amount domestic tea planting. Black tea accounts for more than 80% of all tea imports. It shows that black-tea-related products have become the main tea products consumed by Taiwanese people. Due to the increase in black tea processing and international trade, there have been concerns about the formation of PAHs in tea products. Some studies have focused on the formation mechanism of PAHs [22,23,24] and improving PAHs analysis methods [25,26,27,28]. However, there are still few studies on the dietary exposure and health risks of PAHs from high and long-term consumption of black tea products.

Our previous study conducted background concentration of PAH4 in commercial black tea leaves and infusions in Taiwan [29]. The main aim of this study is to conduct a dietary risk assessment of PAH4 in various black teas and their infusions available on the Taiwan market for the population. In addition, black tea is rich in antioxidants, making it a very popular drink. This study also conducted a risk-benefit analysis to identify black tea products with high levels of polyphenols and low levels of PAHs.

## 2. Materials and Methods

### 2.1. Consumption Data of Tea Infusion and Drinks

The Nutrition and Health Survey in Taiwan (NAHSIT) is a nationwide survey funded by the Taiwan Health Promotion Administration, Ministry of Health and Welfare (former as Department of Health) with the aim to assess the health and nutrition of the Taiwanese population [30]. This survey has been conducted since the 1980s. In 1993 NAHSIT was initiated and included a 24 h dietary recall for Taiwanese individuals aged 13–64 years as well as dietary habit surveys for children aged 4–12 years old between the years of 1993 to 1996. Subsequently, to examine the nutritional status of the population, surveys were conducted for the elderly population between 1990 and 2000, elementary school children in 2001–2002, and children aged 0–6 years and adults aged 19 years and above in 2005–2008. In 2010–2011, surveys were conducted for junior high and high school students, and in 2012, a survey was carried out for elementary school students. The most recent survey was conducted in the year 2013–2016 and included the whole population. The NAHSIT is an annual survey program. The survey results in the year before last are released on official web every year, as well as for updating the National Food Consumption Database.

In 2013, the National Food Consumption Database (NFCD) in Taiwan has also established a four-tier classification framework for Taiwanese dietary intake, consisting of major categories, subcategories, detailed items, and specific items [31]. As of 2018, the NFCD has been using the data from the round of NAHSIT conducted between the years 2013 to 2016. The NFCD operates with the goal of providing reliable food intake data for the population as well as facilitate health risk evaluations.

In this study, the consumption of tea infusion and drinks and body weight of each age group cover aged 6–12, 12–16, 16–18, 19–65, and over 65 years, respectively, was obtained from the NFCD. The above age group data also includes detailed data of the whole group (WG) and consumer only (CO).

### 2.2. Sample Collection, Analysis of the PAH4 in Black Tea Infusion and Drinks

In our previous study, thirty-four black tea samples were obtained from Taiwanese distributors, tea shops and supermarkets [29]. Sixteen of these samples were of Taiwan origin, the other eleven originated from Vietnam (n = 3), India (n = 6), Indonesia (n = 1), Sri Lanka (n = 5), Myanmar (n = 1), and Kenya (n = 2). The preparation of black tea infusion was prepared in a similar way to normal home preparation, wherein 3 g of the steamed leaves was steeped in 150 mL of 85 °C water for 5 min.

The QuEChERS (quick, easy, cheap, effective, rugged, safe) was used to extract PAHs from each sample [29,32]. For tea infusion, 10 mL of the infusion was homogenized. After homogenization, 10 mL of acetonitrile (1% acetic acid) was added and the mixture was homogenized. A QuEChERS extraction salt packet was added to the mixture, which was homogenized for 1 min and centrifuged at 4000 rpm for 5 min. Then, 6 mL of the supernatant was transferred into a QuEChERS column for further clean-up treatment. After being homogenized and centrifuged, 1 mL of the supernatant was collected, filtered through a 0.22 µm PVDF membrane, and used for PAHs analysis.

In this study, high-performance liquid chromatography coupled with fluorescence detection (5000 series, Hitachi, Tokyo, Japan) was used to determine the PAHs in tea. Pinnacle II PAH column (150 × 3.0 mm, 4 µm) (Restek, Bellefonte, PA, USA) was used and kept at 35 °C. The analysis conditions are as follows: mobile phase A (dd H_2_O) and B (ACN:THF, 96:4) (70% B from 0 to 14 min, 90% from 14 to 15 min, 100% from 15 to 27 min, 70% from 27 to 30 min.); flow rate at 1.4 mL/min from 0 min, 2.0 mL/min from 15 to 27 min; injection volume, 20 µL. The excitation wavelengths are from 273 to 302 nm; the emission wavelengths are from 384 to 452 nm [29]. The limit of detection (LOD) and limit of quantification (LOQ) for BaA, CHR, BbF and BaP were 0.02–0.11 µg/kg and 0.08–0.38 µg/kg respectively, which are within the regulations set by the EU [33]. The method validation results demonstrated that our approach was accurate, and precise, ensuring the reliability of the subsequent findings [34].

### 2.3. Handling of Non-Detected Results

According to WHO/ICPS (2009) [35] it should be assumed that a food may contain the chemical of interest even if its quantified amount is lower than the LOD. In this instance, one option would be to assign the ND values based on the LOD. The GEMS/Food-EURO [36] suggested that if ≤60% of the results were below the LOD, the ND should be assigned the value of half the LOD. For results with >60 but ≤80%, the ND would be assigned 0 or LOD. For results with >80%, the ND shall be given the value of 0 or LOD. In addition to this, the use of lower bound (LB) and upper bound (UP) values can be used to calculate estimated dietary exposure, where LB is given the value of 0 and UB is given the value of the LOD.

### 2.4. Dietary Risk Assessment

According to NAHSIT, the main drinkers of tea in Taiwan were aged 6 and above. The population in this study was separated into five groups, those being aged 6–12, 12–16, 16–18, 19–65, and over 65 years. The calculation of the margin of exposure (MOE) has been recommended by the EFSA (2012) [37] as an assessment of impurities that are genotoxic and carcinogenic. The formula for the calculation of MOE is the ratio of the BDML_10_ (benchmark lower dose confidence limit) of the substance and the estimated daily intake (EDI).

In this study, the calculation of MOE is based on the EDI_WG (whole group) and EDI_CO (consumer only). The EDI was calculated for the different age groups of the Taiwanese population by dividing the daily tea consumption rate of each group by their body weight. The estimated PAH intake was then calculated by multiplying this number by the measured concentration of PAH in black tea.

The BMDL is defined as the 95% confidence limit of the BMD (benchmark dose), which is the dose that corresponds to a specific change in an adverse response compared to the response in unexposed subjects. The BMDL_10_ values proposed by the EFSA (2008) [11] were 0.34 mg/kg BW/day for PAH4 and 0.07 mg/kg BW/day for BaP.

According to the EFSA, MOE indicates the level of concern at which results below 10,000 show high levels of concern and results above and furthering away from 10,000 show lower levels of concern. Once the MOE is below 10,000, risk management strategies must be formulated in order to mitigate the risk [37].

$$MOE=\frac{{BMDL}_{j10}}{{EDI}_{i.j}}=\frac{{BMDL}_{j10}}{\left.{\sum_{k=1}^{n}\;\left[C_{k.j}*\left(\frac{{CR}_{k}}{BW}\right)\right.}_{i}\right]}$$

BMDL: benchmark lower dose limit (mg/kg BW/day)

EDI: the estimated average daily intake (mg/kg BW/day)

C: concentration of PAHs in tea (mg/kg)

CR: consumption rate of tea (g/day)

BW: body weight (kg)

### 2.5. Risk–Benefit Assessment

In addition to the exposure assessment of PAHs in black tea, this study also conducted a risk–benefit assessment. Risk–benefit analysis is a process that evaluates the potential risks and benefits associated with a particular activity or product. When applied to the context of food, a risk–benefit analysis examines the potential risks and benefits associated with consuming or producing certain types of food.

The process of conducting a risk–benefit analysis follows a similar framework to that of a risk analysis, encompassing risk–benefit assessment, risk–benefit management, and risk–benefit communication. The risk–benefit assessment phase also follows the steps involved in a risk assessment and includes reduced adverse health effects identification, reduced adverse health effects characterization, exposure assessment, and benefit characterization. Subsequently, the risks and benefits identified are compared in order to make informed decisions and develop appropriate risk–benefit management strategies. Finally, the findings and outcomes of the assessment are effectively communicated to relevant stakeholders to ensure transparency and informed decision-making [38].

Consumption of black tea is often recommended as it contains many beneficial bioactive compounds, one group of these compounds being polyphenols. The daily intake of polyphenol was calculated on the basis of three cups of tea being consumed daily under the assumption that each cup was 150 mL. The values of polyphenols were referenced from a study conducted by Chiang et al. (2021) [39]. This study determined Taiwanese black tea to have an average polyphenol concentration of 236 µg/mL.

## 3. Results

### 3.1. Concentration of PAH4 in Tea Infusion and Drinks

In our previous study, the concentration of PAH4 in black tea leaves ranged from 2.88 µg/kg to 218.21 µg/kg. The concentration of BaP ranged from ND to 47.92 µg/kg. Among the black teas from all seven countries, the lowest concentration of PAH4 was determined in teas which originated in Taiwan. The sum of all 4 PAHs ranged from 2.88 µg/kg to 40.76 µg/kg with a mean sum of 10.12 µg/kg. The mean of BaA, CHR, BbF and BaP were 2.06 µg/kg, 5.20 µg/kg, 0.66 µg/kg, and 2.10 µg/kg respectively. BbF was not detected in many of the Taiwanese tea samples [29].

This study continuously analyzed the tea infusions of the previously studied tea leaves as well as bottled infusions and infusions from tea shops. Among the teas analyzed the infusions from India had the highest concentration of PAH4 with a mean concentration of 0.08 µg/kg. This was followed by the teas from Vietnam with a mean concentration of 0.07 µg/kg. Sri Lanka followed with the third highest concentration of PAH4 with an average concentration of 0.05 µg/kg. The mean concentration of PAH4 in the teas which originated in Taiwan was 0.02 µg/kg. The concentration of PAH4 in all teas originating from Indonesia, Kenya, and Myanmar were determined to be below the LOD.

Similarly to the tea leaves, the tea infusions had higher concentrations of BaA and CHR compared to BbF and BaP. The concentrations of BaA and CHR ranged from ND to 0.21 µg/kg and ND to 0.24 µg/kg respectively. The concentrations of BaP ranged from ND to 0.05 µg/kg. The concentration of BbF was determined to be below the LOD in all infusions analyzed. The concentrations of BaA, CHR and BaP were determined to be below the LOD in majority of the infusions analyzed.

This study also determined the transfer percentage of PAH4 from tea leaves to tea infusions whereby transfer percentages are shown in Table S1. BaA was determined to have the highest transfer, with transfer percentages ranging from ND to 47.13%. This was followed by CHR with transfer percentages ranging from ND to 29.82%. BaP followed with ranges from ND to 0.37%. BbF was not detected in any of the samples analyzed.

### 3.2. Handling of Non-Detected Results

In order to estimate the concentration of PAH4 for the risk assessment for each age group, this study used the suggestions from GEMS/Food-EURO [36] in order to substitute the values according to the percentage of ND. The ND% for BaA, CHR, BbF and BaP were 88%, 94%, 100%, and 85%, respectively. Since the values of ND for each PAH were above 80% the ND would be substituted as 0 or LOD. For this study, the 0 will take on the lower bound (LB) value and the LOD will take on the upper bound (UB) value. Our previous study has confirmed that the LODs of BaA, CHR, BbF, and BaP are 0.1, 0.13, 0.13 and 0.02 (µg/kg), respectively. In addition, based on the ND% and the concentration of detected PAH (Table S1), the average concentrations of each PAH in black tea infusions are shown in Table 1.

### 3.3. Dietary Risk Assessment

#### 3.3.1. Estimated Daily Intake (EDI)

The dietary intake in this study was based on the assumption that all tea products consumed by the public were black tea. To estimate the BaP and PAH4 exposure for all age groups, the estimated daily intake (EDI) was calculated for each group. The calculation of the EDI took into consideration including whole-group (EDI_WG) and consumer-only (EDI_CO) EDIs as well as the lower bound (LB) and upper bound (UB) for non-detected values.

The EDI_CO of BaP from black tea drinks ranged from 0.040 ng/kg BW/day to 0.240 ng/kg BW/day. The 19–65 age group had the highest intake of BaP with an EDI _LB of 0.055 ng/kg BW/day and an EDI_UB of 0.240 ng/kg BW/day. The over 65 years group followed with the second highest intake with an EDI_LB of 0.053 ng/kg BW/day and an EDI_UB of 0.234 ng/kg BW/day. The third highest EDI of BaP was in the 6–12 age group, with an EDI_LB of 0.047 ng/kg BW/day and an EDI_UB of 0.206 ng/kg BW/day. The 12–16 age group had the lowest EDI among all five groups observed. The group had an EDI_LB of 0.040 ng/kg BW/day and an EDI_UB of 0.174 ng/kg BW/day.

In Table 2, the EDI_CO of PAH4 from black tea drinks ranged from 0.287 ng/kg BW/day to 4.369 ng/kg BW/day. The EDI of PAH4 in the consumer-only group follows the same pattern as the EDI of BaP with age 19–65 > over 65 > 6–12 >16–18 > 12–16. The EDI_LB and EDI_UB for the 19–65 age group were 0.395 ng/kg BW/day and 4.369 ng/kg BW/day, respectively. The over 65 age group had an EDI_LB of 0.386 ng/kg BW/day and an EDI_UB of 4.268 ng/kg BW/day. The 12–16 age group had the lowest intake of PAH4 with an EDI_LB of 0.287 ng/kg BW/day and an EDI_UB of 3.169 ng/kg BW/day.

The study also calculated the EDI for the whole group (EDI_WG) which had lower EDI values compared to those of consumer only. The EDI of BaP from black tea drinks ranged from 0.014 ng/kg BW/day to 1.975 ng/kg BW/day. As shown in Table 3, in the whole group, the 19–65 once again had the highest EDI. The EDI_LB and EDI_UB for the 19–65 age group were 0.025 ng/kg BW/day and 0.108 ng/kg BW/day respectively. The 16–18 age group had the second highest EDI, with EDI_LB and EDI_UB values of 0.016 ng/kg BW/day and 0.071 ng/kg BW/day respectively. For the over 65 age group, these values were 0.016 ng/kg BW/day and 0.068 ng/kg BW/day, respectively.

In Table 3, the EDI_WG of PAH4 among the whole group ranged from 0.104 ng/kg BW/day to 1.975 ng/kg BW/day. The EDI of PAH4 from black tea drinks in the consumer group follows a similar pattern as the EDI of BaP, with the highest intake observed in the 19–65 age group, followed by the 16–18 age group, the over 65 age group, the 12–16 age group, and then the 6–12 age group. For the 19–65 age group, the EDI_LB and EDI_UB values were 0.179 ng/kg BW/day and 1.975 ng/kg BW/day, respectively. The over 16–18 age group had an EDI_LB of 0.117 ng/kg BW/day and an EDI_UB of 1.294 ng/kg BW/day. The 6–12 age group exhibited the lowest intake of PAH4, with an EDI_LB of 0.104 ng/kg BW/day and an EDI_UB of 1.147 ng/kg BW/day.

#### 3.3.2. Margin of Exposure (MOE)

The MOE value of a substance is the ratio of its BMDL to its theoretical, predicted, or estimated dose or concentration of human intake. In general, an MOE value above 10,000 shows a low risk. When the MOE value is below the benchmark of 10,000, public concern and proper measures must be taken in order to mitigate the potential risks associated with the substance [37].

In this study, the MOE for each age group was calculated based on the EDI for each group and the BMDL for each compound. The calculation of the MOE took into consideration the consumer-only (MOE_CO) and whole-group (MOE_WG) MOEs as well as the upper bound (UB) and lower bound (LB) values

As shown in Table 4, the MOE_CO for BaP ranged from 292,000 to 17,673,000. The group with the lowest MOE values for BaP was the 19–65 age group with MOE_LB and MOE_UB values of 1,279,000 and 292,000 respectively. This was followed by the over 65 age group with an MOE_LB of 1,309,000 and an MOE_UB of 299,000. The group with the highest MOE for BaP was the 12–16 age group with MOE_LB and MOE_UB values 1,485,000 and 339,000 respectively. The consumer-only MOE for PAH4 follows the same pattern as the MOE for BaP with age 19–65 < over 65 < 6–12 < 16–18 < 12–16. The MOE_LB values for the age 19–16, over 65, 6–12, 16–18 and 12–16 were 861,000, 881,000, 999,000, 1,143,000 and 1,186,000 respectively. The MOE_UB values for the respective groups were 78,000, 80,000, 90,000, 103,000, and 107,000.

The dietary risk assessment of BaP and PAH4 in black tea infusions for the whole group was shown in Table 5. The MOE_WG for BaP ranged from 646,000 to 4,871,000. The group with the lowest MOE for BaP was the 19–65 age group with MOE_LB and MOE_UB values of 2,829,000 and 646,000 respectively. This was followed by the 16–18 age group with MOE_LB and MOE_UB values of 4,319,000 and 986,000, respectively. The group with the highest MOE values was the 6–12 age group with MOE_LB and MOE_UB values of 3,278,000 and 296,000 respectively. The consumer-only MOE for PAH4 follows the same pattern as the MOE for BaP, with age 19–65 < 16–18 < over 65 < 12–16 < 6–12. The MOE_LB values for age 19–65, 16–18, over 65, 12–16 and 6–12 age groups were 1,904,000, 29,007,000, 3,019,000, 3,254,000 and 3,278,000, respectively. The MOE_UB values for the respective groups were 172,000, 262,000, 272,000, 295,000, and 296,000, respectively.

## 4. Discussion

### 4.1. Dietary Risk Assessment

The risk assessment was based on the average concentration of BaP and PAH4 in tea infusions. The MOE was calculated using the benchmark lower-dose confidence limit (BMDL_10_), which is 0.07 mg/kg bw/day for BaP and 0.34 mg/kg bw/day for PAH4. According to EFSA, MOE values near 10,000 or below are considered a health risk concern. Table 6 shows the MOE comparison of various age groups exposed to BaP and PAHs through tea infusions in this study and in Vietnam [40], Poland [41], and South Korea [42]. The MOE values for all age groups were well above the reference number of 10,000 showing that there is little risk to BaP and PAH4 exposure due to tea consumption for the population. While the results indicate a low concern it would be important to monitor and evaluate the levels of PAH in tea regularly. In comparison, only our study explored PAHs exposure to tea drinks among consumers of different age group. In Table 6, the exposure to PAH4 by tea consumption of each age group in Taiwan is higher than that in other countries (MOE values between 172,000–296,000). This also indicates that Taiwanese consumers have a relatively high consumption of tea, especially black tea. Further investigations can prioritize the evaluation of combined exposure sources and analyze the potential cumulative effects they may have. Additionally, it is crucial to assess the long-term health consequences to ensure the ongoing safety of the population (Table 6). Based on the assumption that all tea products consumed by the public are black tea. Even though tea consumption is overestimated, it still shows that the risk of consumers being exposed to PAHs through black tea is low.

### 4.2. Risk–Benefit Analysis

This study conducted a risk–benefit assessment of PAHs in black tea. The assessment followed a framework similar to risk analysis and included steps such as positive health effect identification, benefit characterization, and the comparison between risk and benefits [38].

In addition to PAHs, tea contains many beneficial compounds such as polyphenols. The popularity of tea can be attributed to its numerous beneficial properties. Scientific studies have shown that tea intake can be associated with the reduced risk of cancer, diabetes, and cardiovascular diseases. The presence of polyphenols in tea is believed to contribute significantly to these beneficial health effects. Polyphenols are known to be antioxidants and anti-inflammatories, which may play a key role in the potential health benefits of tea. The risk and benefits end points for PAH4 and polyphenols in tea are summarized in Table 7.

A study conducted by Zamora-Ros et al. (2013) [43] suggested that diets high in polyphenols may be associated with longevity. The study showed that individuals who had polyphenol rich diets, consuming over 600 mg of polyphenol per day, had lower mortality rates compared to those who consumed a lesser amount. Many studies have looked at the levels of polyphenols in tea. According to a study by Chiang et al. (2021) [39], black tea in Taiwan contains an average of 236 µg/mL of polyphenols. Under the assumption that an individual would consume three cups of tea per day, with each cup being 150 mL, the daily intake of polyphenols would be approximately 106,200 µg—or 106.2 mg—per day. In comparison, using the 19–65 age group and PAH4 upper bound value (0.403 µg/kg) as a basis (Table 1), the group would have an EDI of 2.43 ng/kg BW/day.

Under the assumption of three cups per day, the daily intake of polyphenols would be approximately 17.7% of the recommended value stated by Zamora-Ros et al. [43]. By comparing the previously obtained PAH4_EDI value for the adult population with the EFSA-recommended BMDL for PAH4 [11], the MOE value is calculated to be 124,000. This value, being higher than the benchmark of 10,000, indicates a low level of public health concern. Considering the current evidence that tea consumption does not pose a risk to the population, it is reasonable to recommend moderate levels of tea consumption to obtain its potential benefits for health.

The limitations of this study include that due to the use of the 24 h dietary recall method in the NAHSIT database, the collected questionnaires may differ significantly from individuals’ usual dietary habits on that specific day. This could lead to variation in individual dietary intake and result in an overestimation of high-end intake levels in the population. Non-detected values may occur during sample measurement, meaning that the concentration of certain substances is below the LOD. This study set upper bound (UB) and lower bound (LB) limits based on the GEMS/Food-EURO [36] setting principle for the calculation of the exposure assessment. Thus, uncertainty in exposure assessment can be minimized in this study.

## 5. Conclusions

Tea is a significant part of Taiwanese culture. However, the processing of tea may produce PAHs. Therefore, this study aimed to explore the possible health risks due to tea consumption in Taiwan. Assessing the potential health risks posed by PAH consumption, we estimated the daily intake of PAH for different age groups in the population. The 19–65 age group was found to have the highest estimated daily intake. This finding highlights the importance of addressing PAH exposure concerns among this particular population group. This study calculated the MOE for BaP and PAH4. The values were all well above the 10,000 benchmark, and it can be concluded the current levels of PAHs in tea do not pose a risk to the Taiwanese population. From a public health perspective, the benefits of consuming naturally occurring functional components in black tea outweigh the risks of exposure to PAHs. Further research could focus in depth on the factors that influence the concentration of PAHs in tea leaves, and explore the effects of different processing, manufacturing and brewing methods on the PAHs concentration in tea infusions and drinks. Currently, there are no guidelines for BaP or PAH4 in tea products. This study opens up an opportunity for regulatory bodies to begin to implement standards and guidelines for PAHs in tea products.

## Figures and Tables

**Table 1:** The average concentration of PAH4 in tea infusions according to the GEMS/Food LB and UB settings.

Tea Infusions	PAHs (µg/kg)				
	BaA	CHR	BbF	BaP	PAH4
LB	0.017	0.014	0.000	0.005	0.036
UB	0.114	0.137	0.130	0.022	0.403

LB: lower bound, UP: upper bound.

**Table 2:** The lower bound and upper bound estimated daily intake of BaP and PAH4 for consumer only.

Population Group (Age)	BaP		PAH4	
	EDI_CO_LB	EDI_CO_UB	EDI_CO_LB	EDI_CO_UB
	ng/kg BW/day			
6–12	0.047	0.206	0.340	3.763
12–16	0.040	0.174	0.287	3.169
16–18	0.041	0.181	0.298	3.291
19–65	0.055	0.240	0.395	4.369
>65	0.053	0.234	0.386	4.268

EDI: estimated daily intake, CO: consumer only, LB: lower bound, UB: upper bound, BW: body weight.

**Table 3:** The lower bound and upper bound estimated daily intake of BaP and PAH4 for whole group.

Population Group (Age)	BaP		PAH4	
	EDI_WG_LB	EDI_WG_UB	EDI_WG_LB	EDI_WG_UB
	ng/kg BW/day			
6–12	0.014	0.063	0.104	1.147
12–16	0.014	0.063	0.104	1.152
16–18	0.016	0.071	0.117	1.294
19–65	0.025	0.108	0.179	1.975
>65	0.016	0.068	0.113	1.246

EDI: estimated daily intake, CO: consumer only, LB: lower bound, UB: upper bound, BW: body weight.

**Table 4:** Dietary risk assessment of BaP and PAH4 in black tea infusions for consumer only.

Population Group (Age)	BaP		PAH4	
	MOE_CO_LB	MOE_CO_UB	MOE_CO_LB	MOE_CO_UB
6–12	1,485,000	339,000	999,000	90,000
12–16	1,763,000	402,000	1,186,000	107,000
16–18	1,697,000	388,000	1,143,000	103,000
19–65	1,279,000	292,000	861,000	78,000
>65	1,309,000	299,000	881,000	80,000

MOE: margin of exposure, CO: consumer only, LB: lower bound, UB: upper bound.

**Table 5:** Dietary risk assessment of BaP and PAH4 in black tea infusions for whole group.

Population Group (Age)	BaP		PAH4	
	MOE_WG_LB	MOE_WG_UB	MOE_WG_LB	MOE_WG_UB
6–12	4,871,000	1,112,000	3,278,000	296,000
12–16	4,850,000	1,107,000	3,264,000	295,000
16–18	4,319,000	986,000	2,907,000	262,000
19–65	2,829,000	646,000	1,904,000	172,000
>65	4,486,000	1,024,000	3,019,000	272,000

MOE: margin of exposure, WG: whole group, LB: lower bound, UB: upper bound.

**Table 6:** Comparison of BaP and PAH4 MOE_WG_UB between this study and other studies.

Country	Risk Assessment	Population	Results		Reference
Taiwan	MOE	Age 6–12	BaP PAH4	1,112,000 296,000	This study
		Age 12–16	BaP PAH4	1,107,000 295,000	
		Age 16–18	BaP PAH4	986,000 262,000	
		Age 19–65	BaP PAH4	646,000 172,000	
		Age > 65	BaP PAH4	1,024,000 272,000	
Vietnam	MOE	Whole population	BaP PAH4	2,506,928 2,391,604	Phan Thi et al. (2020) [40]
Poland	MOE	Whole population	BaP PAH4	897,000 830,000	Ciemniak et al. (2019) [41]
South Korea	MOE	Whole population	PAH4	4,430,000	Lee et al. (2018) [42]

**Table 7:** Risk and benefits end points for PAH4 and polyphenols in tea.

Type of Effect	End Point	Human Health Relationship
Risk	Kidney disease	Increased risk of kidney disease due to PAH
Risk	Liver disease	Increased risk of liver disease due to PAH
Risk	Cancer	Increased risk of some cancers due to PAH
Benefit	Cancer	Reduced risk of some cancers due to phenolic compounds
Benefit	Heart disease	Reduced risk of heart disease due to flavonoids
Benefit	Diabetes	Reduced risk of diabetes due to catechins

## Data Availability

Data are contained within the article and Supplementary Materials.

## Supplementary Materials

The following supporting information can be downloaded at: https://www.mdpi.com/article/10.3390/toxics12020134/s1, Figure S1: Risk–benefit assessment framework diagram discussed in the EFSA initial proposal [38]; Table S1: Concentration of PAH4 in black tea infusions from various countries.
