# Supplementary material for: Dietary Exposure and Health Risk Assessment of Polycyclic Aromatic Hydrocarbons in Black Tea Consumed in Taiwan
Source: Toxics. 2024 Feb 7;12(2):134. doi: 10.3390/toxics12020134 (PMC10892830; doi:10.3390/toxics12020134)
Supplement: Supplementary file 1 [file toxics-12-00134-s001.zip › toxics-2853859-supplementary.pdf]

toxics

Supplementary Materials: Dietary exposure and health risk assessment of polycyclic aro-matic hydrocarbons in black tea consumed in Taiwan

Drewyan M. Harrison, Wei-Chung Chang and Hsin-Tang Lin

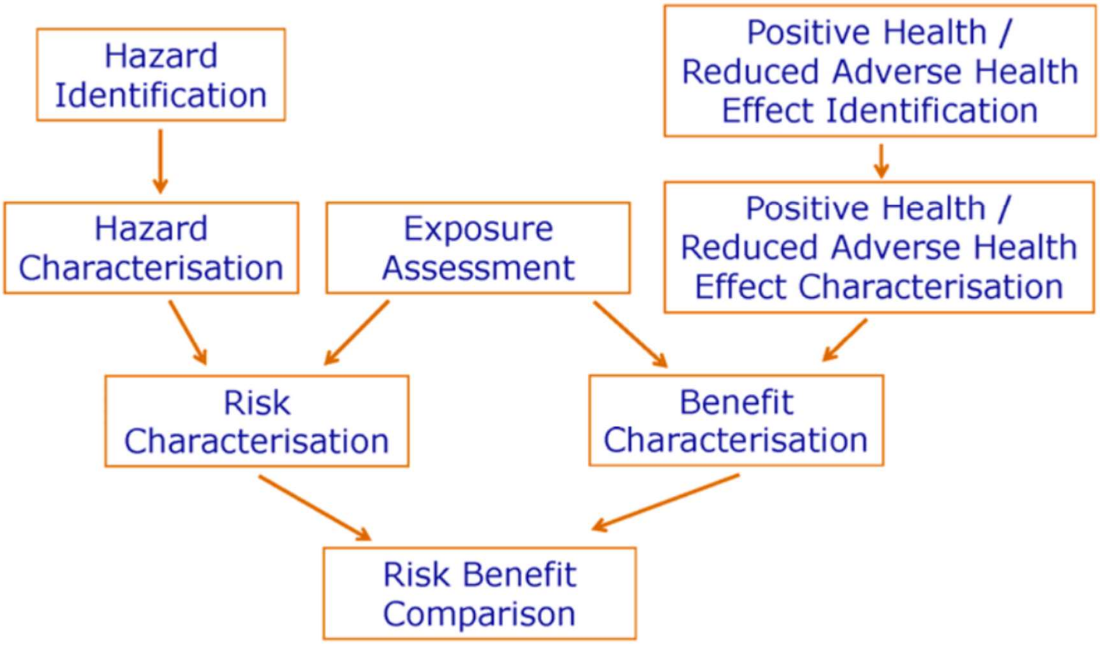

Figure S1. Risk-benefit assessment framework diagram discussed in the EFSA initial proposal [38].

Table S1. Concentration of PAH4 in black tea infusions from various countries.

Country	Sample number	(Group)	Concentration(μg/kg)					Transfer (%)				
			BaA	CHR	BbF	BaP	PAH4	BaA	CHR	BbF	BaP	PAH4
Taiwan	1	(Loose Leaf)	ND*	ND	ND	ND	ND	0.00	0.00	0.00	0.00	0.00
	2	(Loose Leaf)	ND	ND	ND	ND	ND	0.00	0.00	0.00	0.00	0.00
	3	(Loose Leaf)	ND	ND	ND	ND	ND	0.00	0.00	0.00	0.00	0.00
	4	(Loose Leaf)	ND	ND	ND	ND	ND	0.00	0.00	0.00	0.00	0.00
	5	(Loose Leaf)	ND	ND	ND	ND	ND	0.00	0.00	0.00	0.00	0.00
	6	(Loose Leaf)	0.12	ND	ND	ND	0.12	47.13	0.00	0.00	0.00	20.12
	7	(Loose Leaf)	ND	ND	ND	ND	ND	0.00	0.00	0.00	0.00	0.00
	8	(Loose Leaf)	ND	ND	ND	ND	ND	0.00	0.00	0.00	0.00	0.00
	9	(Loose Leaf)	ND	0.24	ND	ND	0.29	0.00	29.82	0.00	0.00	25.81
	10	(Teabag)	ND	ND	ND	ND	ND	0.00	0.00	0.00	0.00	0.00
	11	(Bottle Drink)	ND	ND	ND	ND	ND	-	-	-	-	-
	12	(Bottle Drink)	ND	ND	ND	ND	ND	-	-	-	-	-
	13	(Bottle Drink)	ND	ND	ND	ND	ND	-	-	-	-	-
	14	(Bottle Drink)	ND	ND	ND	ND	ND	-	-	-	-	-
	15	(Tea Shop)	ND	ND	ND	ND	ND	-	-	-	-	-
	16	(Tea Shop)	ND	ND	ND	ND	ND	-	-	-	-	-
Vietnam	17	(Loose Leaf)	0.13	ND	ND	ND	0.13	1.44	0.00	0.00	0.00	0.35
	18	(Loose Leaf)	ND	ND	ND	0.04	0.04	0.00	0.00	0.00	0.37	0.08
	19	(Tea Shop)	ND	ND	ND	0.03	0.03	-	-	-	-	-

Country	Sample number	(Group)	Concentration (μg/kg)					Transfer (%)				
			BaA	CHR	BbF	BaP	PAH4	BaA	CHR	BbF	BaP	PAH4
India	20	(Loose Leaf)	ND	ND	ND	ND	ND	0.00	0.00	0.00	0.00	0.00
	21	(Loose Leaf)	ND	ND	ND	ND	ND	0.00	0.00	0.00	0.00	0.00
	22	(Bottle Drink)	ND	ND	ND	ND	ND	-	-	-	-	-
	23	(Bottle Drink)	ND	ND	ND	ND	ND	-	-	-	-	-
	24	(Loose Leaf)	0.21	0.24	ND	ND	0.45	5.55	3.01	0.00	0.00	3.58

	25	(Bottle Drink)	ND	ND	ND	ND	ND	-	-	-	-	-
Indonesia	26	(Loose Leaf)	ND	ND	ND	ND	ND	0.00	0.00	0.00	0.00	0.00
Kenya	27	(Tea Shop)	ND	ND	ND	ND	ND	-	-	-	-	-
	28	(Loose Leaf)	ND	ND	ND	ND	ND	0.00	0.00	0.00	0.00	0.00
Sri Lanka	29	(Loose Leaf)	ND	ND	ND	ND	ND	0.00	0.00	0.00	0.00	0.00
	30	(Loose Leaf)	ND	ND	ND	0.05	0.05	0.00	0.00	0.00	2.29	0.29
	31	(Tea Shop)	ND	ND	ND	0.03	0.03	-	-	-	-	-
	32	(Bottle Drink)	0.12	ND	ND	0.03	0.15	-	-	-	-	-
	33	(Loose Leaf)	ND	ND	ND	ND	ND	0.00	0.00	0.00	0.00	0.00
Myanmar	34	(Teabag)	ND	ND	ND	ND	ND	0.00	0.00	0.00	0.00	0.00

*ND = Not Detected, < LOD.

38. Assunção, R.; Pires, S.M.; Nauta, M. Risk-Benefit Assessment of Foods. *EFSA J.* **2019**, *17*, 170917. Available online: https://efsa.onlinelibrary.wiley.com/doi/full/10.2903/j.efsa.2019.e170917 (accessed on 31 August 2021).
